# Neurocysticercosis, Meningioma, and Silent Corticotroph Pituitary Adenoma in a 61-Year-Old Woman

**DOI:** 10.1155/2012/340840

**Published:** 2012-12-30

**Authors:** Maria del Pilar Ramirez, Juan E. Restrepo, Luis V. Syro, Fabio Rotondo, Francisco J. Londoño, Luis C. Penagos, Humberto Uribe, Eva Horvath, Kalman Kovacs

**Affiliations:** ^1^Department of Pathology, Hospital Pablo Tobon Uribe and Clinica Medellin, Medellin, Colombia; ^2^Department of Neurosurgery, Hospital Pablo Tobon Uribe, Medellin, Colombia; ^3^Division of Neurosurgery, Universidad de Antioquia, Hospital San Vicente de Paul, Medellin, Colombia; ^4^Department of Neurosurgery, Hospital Pablo Tobon Uribe and Clinica Medellin, Medellin, Colombia; ^5^Department of Laboratory Medicine and Pathology, St. Michael's Hospital, University of Toronto, Toronto, ON, Canada; ^6^Division of Otolaryngology, Clinica Medellin, Medellin, Colombia; ^7^Department of Neurosurgery, Clinica Soma, Medellin, Colombia

## Abstract

We report here the case of a 61-year-old woman who presented with hydrocephalus and cystic and solid lesions in sella turcica, suprasellar areas, and third ventricle. After ventriculoperitoneal shunt she developed cognitive changes and the cystic lesions enlarged. Magnetic resonance imaging (MRI) demonstrated multiple cysts and a solid lesion in the sella and around the anterior clinoid process. With diagnosis of neurocysticercosis she underwent craniotomy. Pathologic examination documented two different lesions: viable and dead cysticerci with inflaming infiltration and a left anterior clinoidal meningioma. At the second surgery, six weeks later via transnasal transsphenoidal approach a silent corticotroph pituitary adenoma was removed which was studied by histology, immunohistochemistry, and electron microscopy. To our knowledge, the occurrence of these three different lesions in the sellar area was not described before.

## 1. Introduction

Cysticercosis is the most frequent helminthic disease of the central nervous system. It is endemic in Latin America, Asia, and Africa and it is frequently diagnosed in immigrant populations all over the world. It develops after ingestion of eggs of *Taenia solium*. The embryo crosses the intestinal mucosa, enters the circulation, and reaches solid organs preferentially the brain and muscles. Neurocysticercosis refers to central nervous system infection by the parasite. It can be parenchymal or extraparenchymal. In the parenchymal form the cysticerci are within the brain parenchyma. In extraparenchymal disease the cysticerci migrate in the cerebrospinal fluid (CSF) to the ventricles, cisterns, subarachnoid space, or spinal cord [1–3].

By altering host defenses the cysticerci are able to survive in the human brain resulting in only minimal host inflammation around them. Eventually the parasite loses its ability to control the host defenses and an inflammatory response leads to the death of the cysticerci. The parasite becomes encased in a granuloma, which either resolves or leads to a scar and calcification. There is a wide variability in the immunological reaction of the host as well as in the multiple lesions induced by the parasites [1].

## 2. Case Report

**Figure 1:**
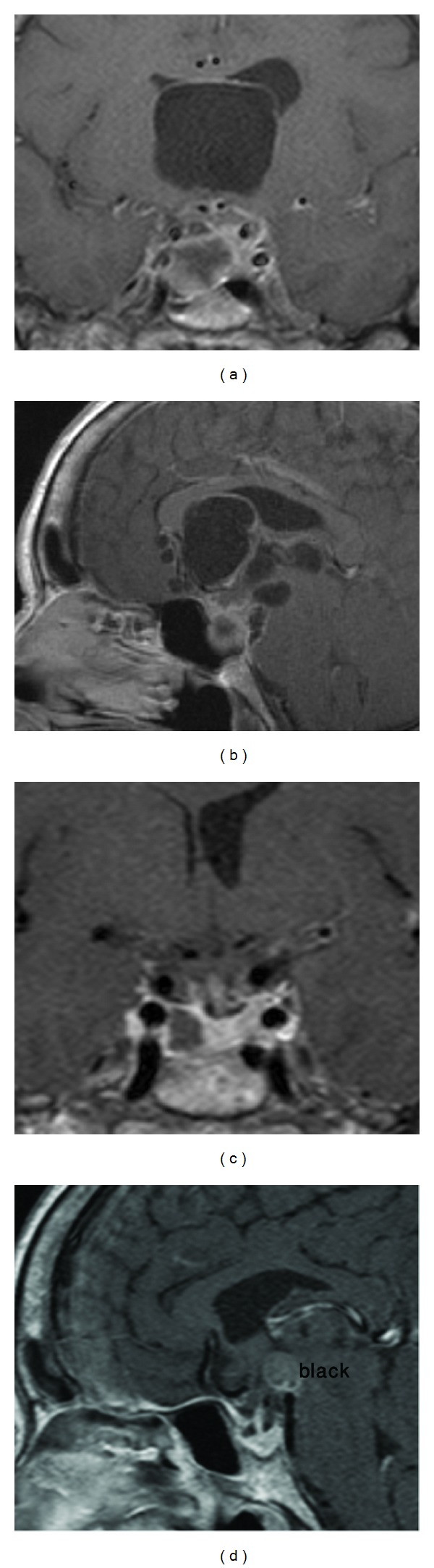
Preoperative. Coronal (a) and sagittal (b) T1-weighted MRI with gadolinium enhancement showing multiple cysts in the sellar, suprasellar regions, third ventricle, and basal cisterns. There is heterogenous contrast enhancement around the left anterior clinoidal process and in the sella turcica. Postoperative coronal (c) and sagittal (d) T1-weighted MRI with gadolinium enhancement showing partial empty sella, resection of the pituitary adenoma, anterior clinoidal meningioma, and the cysts.

A 61-year-old woman presented with two months history of headache and acute hydrocephalus with small cystic lesions in the skull base and intracranial calcifications as documented by MRI. A ventriculoperitoneal (VP) shunt was inserted and the clinical diagnosis of neurocysticercosis was made. She received albendazol for three weeks. 6 months later she developed severe cognitive decline, gait disturbances, and urinary incontinence. She complained of anorexia, malaise, and fatigue. At physical examination, visual fields showed no abnormalities and were normal. Hematological and biochemical studies revealed no changes. Hormone blood levels were growth hormone (GH) 0.32 ng/mL (<2), IGF-1 142 ng/mL (100–295), prolactin 41.4 ng/mL (2–15), luteinizing hormone (LH) 19.8 mIU/mL (40–104), follicle-stimulating hormone (FSH) 34 mIU/mL (34−96), thyroid stimulating hormone (TSH) 1.87 mU/mL (0.5–5), freeT4 1.35 ng/dL (0.7−2), and cortisol 19.2 mcg/dL (3−25). Computed tomography (CT) and magnetic resonance imaging (MRI) scan disclosed multiple intracranial cysts in the suprasellar area with extension to the third ventricle. The sella was enlarged and occupied by a mixed lesion with nonhomogeneous contrast enhancement (Figures 1(a) and 1(b)). It was presumed that the lesions represented cysticercosis in different stages. The patient underwent right frontotemporal craniotomy and several cysts were removed. In some areas there were soft, necrotic material and in others there were viable cysts. Surprisingly a left anterior clinoidal meningioma of 10 × 10 mm was found and resected as well. At one week after operation she developed seizures and CSF examination disclosed meningitis. She was treated with vancomycin, meropenem, and albendazol with good evolution. MRI disclosed persistence of the intrasellar lesion and one month later a microsurgical transnasal endoscope-assisted approach was performed and pituitary tumor was found and removed. The postoperative period was uneventful and she was discharged two weeks later. MRI showed adequate resection of the multiple lesions (Figures 1(c) and 1(d)). Her followup was uneventfully and recent MRI disclosed minimal residual lesions.

## 3. Pathology

**Figure 2:**
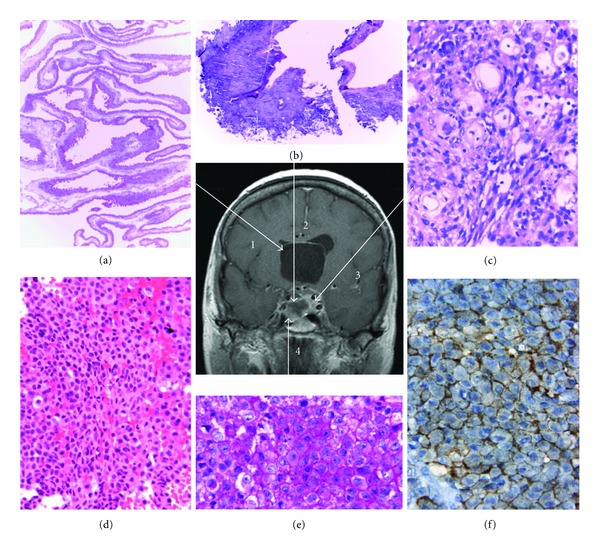
Correlation of MRI and pathological findings. Arrow 1: (a) viable cysticercus cyst (vesicular stage). H&E. Original magnification: 100x. Arrow 2: (b) nonviable cysticercus (granular and calcified stages). H&E. Original magnification: 100x. Arrow 3: (c) Psammomatous meningioma. H&E. Original magnification: 100x. Arrow 4: (d) pituitary adenoma. H&E. Original magnification: 250x; (e) pituitary adenoma. PAS. Original magnification: 400x; (f) pituitary adenoma, ACTH immunostaining. Original magnification: 400x.

The surgically removed tissues were fixed in buffered formalin and embedded in paraffin. From the first surgery one part represented the wall and epithelial lining of a simple cyst contaminated with the parasite compatible with neurocysticercosis in vesicular stage (Figure 2(a)). Other portions revealed necrotic tissue with calcification and lymphocytes corresponding to granular and calcified nodular cysticercosis (Figure 2(b)). A third portion disclosed a benign psammomatous meningioma (Figure 2(c)).

The lesion removed at the second surgery revealed an amphophilic conclusively PAS positive pituitary adenoma with a diffuse pattern (Figures 2(d) and 2(e)). Immunohistochemical studies (streptavidin-biotin-peroxidase complex method) demonstrated immunopositivity for ACTH in the tumor cells (Figure 2(f)). Immunostainings for GH, PRL, TSH, FSH, LH, and alpha subunit were negative. Many tumor cells showed Crooke's hyalinization. The Ki-67 nuclear labeling index using the MIB-1 antibody was less than 1% and MGMT yielded negative results in many tumor cell nuclei. The tumor and a small portion of adjacent nontumorous adenohypophysis were not infiltrated by cysticerci. Electron microscopy demonstrated a variable granulated adenoma consisting of closely apposed small rounded cells. The Golgi apparatus was prominent with many forming secretory granules. The ovoid-rod-shaped-notched irregular and heart-shaped mainly peripherally localized secretory granules were in the 150–313 nm range (mean 225 nm). Bundles of cytokeratin filaments were regularly noted. The diagnosis of silent corticotroph adenoma subtype II was made. 

## 4. Discussion

Cysticerci consist of two parts, the vesicular wall and the scolex. After entering the central nervous system, they are in a viable stage (vesicular stage) with a transparent membrane, a clear vesicular fluid, and an invaginated scolex. They may remain viable for many years or enter in a process of degeneration. The first stage of involution is the colloidal stage, in which the clear vesicular fluid becomes turbid and the scolex suffers hyaline degeneration. Thereafter the wall of the cyst thickens and the scolex is transformed into mineralized granules (granular stage) which are no longer viable. At the end the parasite remnants appear as a calcified nodule (calcified stage) [1, 4]. The inflammatory response elicited by the vesicular stage is minimal but in the colloidal stage they are surrounded by a collagen capsule and mononuclear inflammatory reaction that includes the parasite itself. The surrounding brain parenchyma suffers astrocytic gliosis, microglial proliferation, edema, neuronal degenerative changes, and perivascular infiltration of lymphocytes. When the parasite enters into granular and calcified stages the edema disappears but the astrocytic changes become more intense and epithelioid cells appear forming multinucleated giant cells [5]. The inflammation elicited by meningeal cysticerci is more severe with formation of exudates of collagen fibers, multinucleated cells, lymphocytes, eosinophils, and hyalinized parasitic membranes in the subarachnoid space which lead to leptomeningeal thickening. This inflammation may induce damage to the optic chiasm and cranial nerves, arteritis, cerebral infarction, or obstructive hydrocephalus [1]. The pathogens are accompanied by inflammatory infiltration of variable severity. Some cysticercus antigens stimulate the production of specific antibodies while others play a role in the evasion of the immune surveillance against the parasite. It has been suggested that the cellular immune dysfunction in patients with neurocysticercosis may be associated with oncogenesis and glioma development [6–8].

The presence of cysticerci in the sella is a rare occurrence. Sheehan and Summers [9] in their classical study of hypopituitarism quote the first case reported in 1915 [10]. A man with several symptoms was found at postmortem to have a cysticercus which had destroyed the pituitary gland. After that it was reported as a radiographic [11], autopsy [12], or surgical finding [13–18]. To the best of our knowledge, the occurrence of 3 different lesions in the sella turcica was not described before. Obviously, this is a great challenge to clinical endocrinologists and to those who evaluate imaging results. In our case, the radiologic, CT scan and MRI findings failed to provide a definitive conclusion of the associated lesion and detailed pathologic examination was necessary to reach the proper diagnosis [19]. Albendazol and praziquantel are used for medical treatment and surgery is indicated in case of hydrocephalus, subarachnoid, or intraventricular lesions. Large surgical series had been published showing conventional approaches [20, 21], and recently, minimal invasive flexible endoscopy surgery [22, 23] has been proposed.

A question may arise whether the 3 lesions which are very different in pathogenesis, clinical behavior, imaging findings, and morphology are causally related or are completely independent. As far as we know from the available literature, there is no indication to suggest that one of these lesions played a role in the development of the other lesions and it is not likely that any connection exists. Two different tumors, such as metastatic carcinoma and pituitary adenoma, or two different subtypes of pituitary adenomas may be present simultaneously but they are considered as an accidental event [24]. 

Recent evidence indicates that inflammation may contribute to the development and progression of various tumors. Inflammatory cells may synthesize and release various cytokines which may affect endothelial function, vascular permeability, and the genetic profile of the tumor cells. Thus, the possibility in our case that the inflammation contributed to the development and/or progression of one of the documented tumors cannot be excluded. However, we have no evidence to suggest this possibility.
